# Self-reported vaccination in the elderly: SABE Bogota study, Colombia

**Published:** 2016-03-30

**Authors:** Carlos Cano Gutierrez, Carlos Reyes-Ortiz, Miguel German Borda, Antonio Arciniegas

**Affiliations:** 1 Instituto de Envejecimiento, Facultad de Medicina, Pontificia Universidad Javeriana, Bogota Colombia; 2 UTHealth, The University of Texas Health Science Center at Houston, Department of Internal Medicine, Division of Geriatric and Palliative Medicine, Houston, TX, USA; 3 Unidad de Geriatría, Hospital Universitario San Ignacio. Bogotá Colombia; 4 Semillero de Neurociencias y Envejecimiento, Facultad de Medicina, Pontificia Universiadad Javeriana, Bogotá Colombia

**Keywords:** Aged, vaccination, influenza, pneumococcal vaccines, tetanus, epidemiologic studies

## Abstract

**Objectives:**

To determine the frequency of vaccination in older adults within the city of Bogotá and to estimate the association with sociodemographic and health factors.

**Methods:**

This is a secondary data analysis from the SABE-Bogotá Study, a cross-sectional population-based study that included a total of 2,000 persons aged 60 years. Weighted percentages for self-reported vaccination [influenza, pneumococcal, tetanus] were determined. The association between vaccination and covariates was evaluate by logistic regression models.

**Results:**

A total of 73.0% of respondents received influenza, 57.8% pneumococcal and 47.6% tetanus vaccine. Factors independently associated with vaccination included: 1- age (65-74 years had higher odds of receiving vaccinations, compared to 60-64 years); 2- socioeconomic status (SES) (higher SES had lower odds of having influenza and pneumococcal vaccines, compared to those with lower SES); 3- health insurance (those with contributive or subsidized health insurance had higher odds (between 3 and 5 times higher) of having vaccinations, compared to those with no insurance); 4- older adults with better functional status (greater Lawton scores) had increased odds for all vaccinations; 5- older adults with higher comorbidity had increased odds for influenza and pneumococcal vaccinations.

**Conclusion:**

Vaccination campaigns should be strengthened to increase vaccination coverage, especially in the group more reticent to vaccination or vulnerable to reach it such as the disabled elder.

## Abstract (es)

**Objetivos:**

Determinar la frecuencia de vacunación en la población adulta mayor de la ciudad de Bogotá, y estimar la asociación con factores sociodemográficos y de salud.

**Métodos:**

Este es un análisis secundario de datos del estudio SABE-Bogotá, un estudio poblacional transversal que incluyó un total de 2,000 personas mayores de 60 años. Se obtuvieron porcentajes ponderados del auto-reporte de vacunación (influenza, neumococo, tétano). La asociación entre la vacunación y otras variables se evaluó mediante modelos de regresión logística.

**Resultados:**

el 73.0% se vacunó contra la Influenza, 57.8% contra el Neumococo y el 47.6% recibió la antitetánica. Los factores que estaba independientemente asociados con la vacunación incluyen: 1-edad (65-74 tenían probabilidad aumentada comparada con 60-64 años); 2- nivel socioeconómico (NSE) (mayor NSE tenían menos probabilidades de tener vacunas contra la influenza y neumococo, en comparación con aquellos con menor NSE); 3- seguro de salud (con seguro contributivo o subsidiado tenían probabilidades más altas, (entre 3 y 5 veces mayor) de tener vacunación contra la influenza, neumococo y tétanos, en comparación con aquellos que no tienen seguro); 4- aquellos con estado funcional mejor (puntaje mayor Lawton) tenían probabilidades mayores para todas las vacunas; 5- aquellos con morbilidad aumentada tenían probabilidades mayores para tener vacunas de influenza y neumococo.

**Conclusión:**

Las campañas de vacunación se deben fortalecer para incrementan la cobertura, especialmente en el grupo más reacio a la vacunación o vulnerable para alcanzarla tales como los adultos mayores con discapacidad.

## Introduction

Elderly population has increased rapidly worldwide over the last decades due to the demographic transition 1. Until now life expectancy has doubled and in 2100 it is projected to triple 2. Population aging has increased the demand for health services by older adults. Because of their decreased physiological reserve, they are prone to present more diseases 3. Thus, creating the need for cost-effective public policies that protect older adults. Prevention using vaccines has been an effective measure in increasing survival of millions of people around the world. Immunization in older adults has just been developed during the last decade. This development has derived from a better understanding of immunosenescence, the central theory that explains the age related changes of the immune system, including the increased susceptibility to infections with longer duration and severity, atypical presentations of diseases, lower response to immunization and a higher prevalence of neoplasia 3, 4.

The Center for Disease Control and Prevention (CDC) of the United States, in their vaccination schedule for 2013, recommends that people over 60 should be vaccinated against influenza, Diphtheria, Pertussis, Tetanus (DPT) and, Herpes Zoster and in individuals over 65 years also against Pneumococcus 5. However, the reported CDC vaccination rates still remain low. Coverage for vaccination among individuals of 65 years and older is 59.2% for influenza, 59.9% for Pneumococcus and 55.1% for tetanus, and these rates still remain below target rates 6. Despite the increase in vaccination against pneumococcus between 1997 and 2005, 25% of older adults still reported they never received this vaccine 7. Studies in Latin America, reported that older adults learned about vaccination programs through ads in mass media such as television and radio, whereas only 5% received this information from a physician or healthcare provider. Additionally, 83% of those who were not vaccinated reported that the main reason for avoiding vaccination was fear of potential side effects or because they believed that it would not work 8. 

This is important because although the infection with influenza virus is usually self-limited, in the elderly it may have serious complications. About 90% of deaths caused by influenza occur in individuals over 65 years, often secondary to pneumonia, in which the main risk factors are increasing age and exacerbation of cardiopulmonary diseases 9, 10. Individuals over 85 years with influenza and pneumonia have 32 times higher risk of death, and if they have influenza and any other comorbidity the risk of death is 16 times higher 10. In Colombian, respiratory diseases are the third cause of death among older adults between 65 and 75 years, and the second cause of death in those over 80 years. In most cases, these clinical complications are secondary to infection and are preventable 11. 

Similarly, respiratory pneumococcal disease is a major cause of death in the elderly and its main complication is pneumonia, with mortality rates of 20% in those over 65 years and 40% for those over 85 years 12. There is no evidence suggesting that pneumococcal vaccine prevents pneumonia, however, evidence suggests it decreases the severity of the infection and the probability of pneumococcal bacteremia that can result in multiple diseases such as meningitis. Additionally, pneumococcal vaccination reduces the risk of pneumococcal bacteremia by 44% reduction of the risk 13. 

In addition, tetanus is a serious disease, which presents with mortality rates of up to 50% 14, 15. This is especially important in third world countries, which often have high incidence of tetanus 14. Although in Colombia, we have had a significant reduction, there are still an elevated number of cases and a substantial underreport 15. According to the *Instituto Nacional de Salud de Colombia*, however, between 2000 and 2002, 28 cases of tetanus were reported 16. 

In Bogota, capital city of Colombia, there were more than 7.5 million inhabitants in 2011, of which 10.7% were persons aged 60 years and older 17, 18. Influenza, pneumococcal and tetanus vaccines are available free of cost for persons 60 years and older in Bogota, Colombia 17, 18. 

The objectives of this study were to determine the frequency of vaccination and to estimate the association with sociodemographic and health factors in a sample of older adults in the city.

## Materials and Methods

This report is a secondary data analysis from the *Salud Bienestar y Envejecimiento* (SABE) Bogotá Study, a cross-sectional survey conducted during December of 2012. The SABE Bogotá Study was designed using a probabilistic sampling scheme by clusters (housing segments) with block stratification. A total of 2,000 adults over 60 years of age were interviewed and assessed in their homes. All respondents were community dwelling, and the sample was representative of urban and rural areas of the city, 81.9% of eligible adults agreed to participate in the study. Expanding the survey sample of the SABE study Bogotá, based on population projections for 2012 adjusting by a correction factor by age and sex, the population estimate was of 779,534 older adults 19. 

The instrument used in the SABE Bogotá study was derived from the international instrument designed by the Pan American Health Organization for the Latin American countries including the most relevant topics to this analysis 20: 1) personal and family data (with sociodemographic data) 2) health status (with self-reported vaccination and comorbidity data) 3) functional evaluation. The instrument was modified and adapted to Colombia's context 19. The research protocol was approved by the IRB at Pontificia Universidad Javeriana. An informed consent was signed by all the study participants.

###  Variables 

**Dependent variables**. Vaccination was estimated by calculating the frequencies of self-reported pneumococcal (if have ever had), influenza (in the last year) and tetanus (in the last 10 years) vaccinations. 

**Independent variables. ** Demographic characteristics were described and categorized in groups of age (60-64, 65-69, 70-74 and ≥75 years), sex, socio-economic status, health insurance, functional status, and comorbidity.

Socio-economic status in Colombia is measured using six categories from 1 the lowest, to 6 the highest. For the current study, we grouped the six strata into three groups: 1-2 (lower), 3-4 (middle) and 5-6 (upper). This was done following the methodology reported by the *Departmento Administrativo Nacional de Estadistica* (DANE) 21. Health insurance was categorized based on the social security health system of Colombia, which offers coverage to all citizens and foreign people in Colombia. It has two affiliation systems: 1) Contributive and 2) Subsidized. And for those that are not included within these two categories, they are categorized as "linked to the system", known as a transitory category within the system affiliation, and individuals not insured. Their health coverage is granted by "health service institutions contracts" between territorial entities and government institutions. Functional status was assessed using the Lawton Scale, which measures functionality regarding instrumental activities of daily living (IADL) such as using the telephone, taking medications, managing finances, preparing meals, shopping, housekeeping, doing laundry, using transportation 22. We used binary scores for each question (capable without problems or incapable) and created a total score from 0 to 8, higher scores indicate more functionality. Comorbidity included history of seven medical conditions: hypertension, diabetes, coronary heart disease, arthritis, stroke, chronic pulmonary obstructive disease or cancer. Respondents were asked: "Has a doctor or a nurse told you that you have...?" for each of the conditions previously listed.

### Statistical analyses 

To adjust for sampling survey design, data were weighted by using complex survey analyses. To describe characteristics of study population, categorical variables are presented with percentages whereas continuous variables are presented with means ± standard errors. Frequencies of vaccination by sociodemographic factors or Spearman correlation coefficients by functional status or comorbidity were reported, and differences were tested by the Wald Chi-square or correlation analyses. For the multivariate analyses, weighted logistic regression models were used to test the association of independent variables with vaccination (yes= 1, no= 0), obtaining Odds Ratios (ORs) with 95% confidence intervals (CI). Data were analyzed using SAS^®^ version 9.3 for windows (SAS Institute, Cary, North Carolina-USA). The statistical level of significance was set at *p* <0.05.

##  Results

### General demographic data 

Of the 2,000 individuals interviewed for our study, a total of 62.5% were women, with a mean age of 71.1 ± 0.2 years. About a third were individuals aged 75 and older. Socioeconomic status showed the highest proportion in the lower social (45.6%) and middle class (45.9%), and a lower proportion in the upper social class (8.5%). Regarding health insurance, the higher proportion were affiliated with contributive (72.5%) (Table 1).

**Table 1.** Characteristics of the study population. Persons aged 60 years and older, Bogotá, Colombia.

Category	n (%) or Mean ± SE
Sex	
Women	1,249 (62.5)
Age (years) (range 60-100)	
Mean number	71.1 ± 0.2
60-64	506 (26.0)
65-69	454 (22.9)
70-74	398 (18.8)
≥75	642 (32.3)
Socio-economic status	
Lower class (1-2)	1,038 (45.6)
Middle class (3-4)	897 (45.9)
Upper class (5-6)	65 (8.5)
Health insurance	
Contributive	1,366 (72.5)
Subsidized	571 (24.6)
Transitory	17 (0.8)
Not insured	44 (2.1)
(Functional Status Score in Lawton test, IADLs)	
Mean score (0-8)	7.1 ± 0.0
Comorbidity (Number of diseases)	
Mean score (0-7)	1.4 ± 0.0

Data are weighted.

SE= standard error.

IADLs= instrumental activities of daily living.

Comorbidity includes: hypertension, diabetes, coronary heart disease, arthritis, stroke, chronic pulmonary obstructive disease or cancer

### Vaccination data 

Data showed that 73.0% of the population had received vaccine for influenza during the last year, 57.8% have ever had vaccine for pneumococci, and 47.6% for the tetanus during the last 10 years. 

In bivariate analyses (Table 2), individuals aged 65 to 69 and 70 to 74 years had higher percentages of influenza, pneumococcus, and tetanus vaccination, compared to those between 60-64 years. No gender differences were found. Older adults with higher SES (Upper class) had lower vaccination percentages for influenza and pneumococcus, compared to those with lower SES, (lower and middle class). Individuals covered by health insurance (contributive, subsidized, or transitory) had higher vaccination percentages. There were significant positive correlations between Lawton scores and all three vaccinations. There were significant positive correlations of comorbidity with influenza and pneumococcal vaccinations.

**Table 2.** Vaccination percentages and correlations coefficients for Influenza, Tetanus y Pneumococci, according to sociodemographic and health characteristics. Persons aged 60 years and older, Bogotá, Colombia.

Characteristics	Influenza	Pneumococci	Tetanus
	(%)	(%)	(%)
Vaccinated individuals, n=2,000	73.0	57.8	47.6
Age (years)			
60-64	62.9	48.1	40.6
65-69	76.1	64.5	53.9
70-74	80.5	69.0	52.4
75+	74.6	54.6	45.9
*p*-value	<0.001	<0.001	0.008
Sex			
Women	73.6	58.1	45.9
Men	71.9	57.5	50.4
*p*-value	0.565	0.840	0.144
Socio-economic strata			
Lower class (1-2)	76.6	58.6	49.4
Middle class (3-4)	76.1	63.3	47.9
Upper class (5-6)	36.9	24.3	35.6
*p*-value	<0.001	<0.001	0.211
Health insurance			
Contributive	72.9	59.5	49.1
Subsidized	75.3	55.4	46.1
Transitory	78.8	63.1	42.1
Not insured	44.7	23.8	17.9
*p*-value	0.046	0.004	0.007
Functional Status. Lawton test (IADLs)			
Continuous score (0-8)	0.06*	0.09*	0.13*
*p*-value	0.003	<0.001	<0.001
Comorbidity. Number of diseases (0-7)	0.09 *	0.07 *	0.02 *
*p*-value	<00.001	0.001	0.454

Percentages are weighted.

Differences are tested by the Wald-Chi-square and correlation analyses.

IADLs= instrumental activities of daily living.

Comorbidity includes: hypertension, diabetes, coronary heart disease, arthritis, stroke, chronic pulmonary obstructive disease or cancer.

*Speraman r

In multivariate analyses (Table 3), individuals aged 65 to 69 and 70 to 74 years had higher odds for influenza, pneumococcus, and tetanus vaccination, compared to those between 60-64 years. Older adults with upper class had lower odds for influenza and pneumococcus vaccination, compared to those with lower class. Individuals covered by health insurance (contributive or subsidized) had higher odds (between 3 and 5 times higher) for influenza, pneumococcus and tetanus vaccination, compared to those with no insurance. Older adults with better functional status (higher Lawton scores) had higher odds for influenza, pneumococcus and tetanus vaccinations. Those with higher number of medical conditions (comorbidity) had higher odds for influenza, and pneumococcus vaccinations.

**Table 3.** Factors associated with vaccination, weighted multivariate logistic regression analyses, n= 2,000.

Characteristics	Influenza		Pneumococci		Tetanus	
	OR (95% CI)	*p*-value	OR (95% CI)	*p*-value	OR (95% CI)	*p*-value
Age (years)						
60-64	1.00		1.00		1.00	
65-69	1.77 (1.22-2.56)	0.002	1.88 (1.34-2.64)	<0.001	1.68 (1.17-2.39)	0.004
70-74	2.04 (1.35-3.07)	<0.001	2.10 (1.43-3.08)	<0.001	1.63 (1.12-2.36)	0.009
75+	1.74 (1.15-2.62)	0.007	1.33 (0.95-1.86)	0.096	1.48 (1.03-2.12)	0.032
Sex						
Men	1.00		1.00		1.00	
Women	1.14 (0.86-1.53)	0.354	1.06 (0.82-1.36)	0.653	0.85 (0.66-1.09)	0.217
Socio-economic strata						
Lower class (1-2)	1.00		1.00		1.00	
Middle class (3-4)	0.89 (0.67-1.18)	0.426	1.10 (0.85-1.42)	0.463	0.87 (0.67-1.11)	0.269
Upper class (5-6)	0.16 (0.08-0.30)	<0.001	0.20 (0.10-0.38)	<0.001	0.50 (0.24-1.03)	0.060
Health insurance						
Not insured	1.00		1.00		1.00	
Contributive	3.47 (1.65-7.32)	<0.001	4.84 (2.18-10.74)	<0.001	4.55 (2.11-9.83)	<0.001
Subsidized	3.00 (1.39-6.45)	0.005	3.58 (1.60-8.01)	0.002	3.70 (1.69-8.07)	0.001
Transitory	3.76 (0.93-15.25)	0.063	5.11 (1.22-21.37)	0.025	3.32 (0.77-14.25)	0.105
Functional Status. Lawton test (IADLs)						
Continuous score (0-8)	1.13 (1.03-1.23)	0.010	1.08 (1.00-1.17)	0.048	1.11 (1.02-1.20)	0.012
Comorbidity. Number of diseases (0-7)	1.23 (1.08-1.39)	0.002	1.16 (1.04-1.30)	0.007	1.02 (0.91-1.14)	0.691

OR= odds ratios

CI= confidence intervals.

IADLs= instrumental activities of daily living.

Comorbidity includes: hypertension, diabetes, coronary heart disease, arthritis, stroke, chronic pulmonary obstructive disease or cancer

## Discussion

Having assessed the coverage of vaccinations in elders living in the city of Bogota and having identified factors that influenced the likelihood of being vaccinated are the most important results of this report.

In the current report the vaccination weighted percentages were 73.0% for influenza, 57.8% for Pneumococci, and 47.6% for tetanus which corresponds, respectively, to a) 569,060, b) 450,571 and c) 371,058 persons aged 60 years and older, living in the city of Bogotá in 2012. Our result on influenza vaccination is comparable to one study in Sao Paulo, Brazil, in 2010, where adults aged 60 and older had a self-reported vaccination of 74.2% 23.

**Figure 1.**
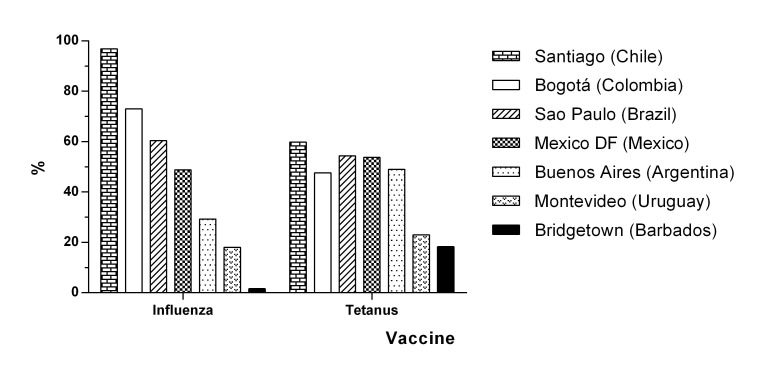
Vaccination for Influenza and Tetanus in Bogota and other cities in Latin America, persons aged 60 years and older. Data are weighted percentages of influenza vaccine during the previous year and tetanus vaccine during the previous 10 years, from the SABE Study in Latin American cities and the SABE Bogota Study.

Our results could be also comparable, in part, with data from another secondary analysis of a study with similar design to the SABE Bogota, the Latin American SABE Study in 1999-2000, including persons 60 years and older from 6 cities: Bridgetown (Barbados), Montevideo (Uruguay), Buenos Aires (Argentina), Mexico City (Mexico), Sao Paulo (Brazil), and Santiago (Chile). The weighted percentages of influenza vaccine during the previous year and the weighted percentages of tetanus vaccine during the previous 10 years are depicted in Figure 1. Herein, Bogota has higher percentage for influenza vaccine than those cities, except Santiago. However, Bogota has lower percentages for tetanus vaccine compared to Santiago, Sao Paulo and Mexico City 24.

Similarly to our results, older adults residing in Buenos Aires, Sao Paulo and Mexico City with any health insurance were significantly more likely to have influenza vaccine (OR= 2.21, 95% CI= 1.41-4.07; OR= 4.64, 95% CI= 2.33-9.22; OR= 2.21, 95% CI= 1.64-2.99; respectively) and tetanus vaccine (OR= 1.70, 95% CI= 1.03-2.80; OR= 1.98, 95% CI= 1.06-3.71; OR= 2.19, 95% CI= 1.63-2.96; respectively) compared to individuals with no health insurance 24. 

One study designed to determine vaccination coverage among persons aged 60 and older, done in Mexico by Trejo-Valdivia *et al*. in 2008, showed that national vaccination coverage for influenza was 56.5%, for pneumococci was 44.3% and for tetanus was 61.8%. 25 However, that study differs from our study in the following: First, it was designed to specifically determine vaccination coverage (our report is a secondary analysis of the SABE Bogota study which covers multiple health problems and diagnoses), and in addition to self-report they compared the information provided by the elderly with vaccination health records or carnets. Second, the tetanus vaccine question asked if a person had it at 60 years or older (not during the previous 10 years as it was in our study) 25. 

Our finding that older adults with functional impairments are less likely to have a vaccine is in agreement with a previous report on influenza and tetanus vaccines 24. Disabled individuals may have barriers to go on time to the health care settings or to be involved in vaccination campaigns. Thus, disable older adults are important health promotion targets to increase vaccination coverage in Bogota and other cities in Colombia.

In our study, older adults with more comorbidity have greater vaccination for influenza and pneumococci. This finding is in line with previous reports 26,27. Indeed, older adults with chronic conditions are more likely to have attended health care settings where additional advice for vaccination may have been provided; especially the direct recommendation by a healthcare provider plays a decisive role in the decision to have a vaccine 26,27. 

It could be argued that the efforts made by health centers (after the pandemic outbreak of H1N1 in 09), for raising awareness about the importance of vaccination have been successful. However, it would be fair to acknowledge that it also was the result from a long-time work carried out by different academic and social organizations that have historically pursued the same target 28. Although the results have been good, they are not sufficient and we are still far from universal coverage for this population. On the other hand, tetanus vaccination has not been sufficiently publicized as compared to the other two vaccines, which could explain the lower coverage of the tetanus vaccine.

Women usually have greater acceptance and better adherence towards preventive programs, but in our study we did not find sex differences 29. As expected, individuals with health insurance affiliation had higher coverage of all vaccines, both in the contributive and subsidized category compared to those that reported no affiliation. Interestingly enough, the transition group had the highest prevalence of pneumococcal vaccination, which is not the case for the other vaccines. This finding maybe explained by the extensive free vaccination campaigns for older adults, carried out in the city during the last decade, but still not reaching people without insurance. It is surprising the poor acceptance of these preventive programs in the upper social classes, which would suggest that prevention campaigns have reached more impact in the lower and middle social class. It should be taken into account that the questions related to the pneumococcal vaccine, did not ask about the specific time period of the shot.

Our study has some limitations. First, this is a cross-sectional study. Therefore, causality cannot be determined. Second, self-reported vaccinations are used as the outcome variables, and we did not corroborate the self-report with medical or administrative records, or vaccination carnets. This is not exempt of recall bias (e.g., over reporting), which may lead to analytical bias. Nevertheless, our study has some strength. The design of this study was planned to allow statistical inference for the population aged 60 years and older. Thus, we are reporting weighted frequencies for self-reported vaccination in a representative sample of older adults in the capital city of Colombia, Bogota.

Individuals 65 to 74 years had the highest percentages of vaccination compared with the younger group between 60 to 64 years. Thus, improving public health policies within this younger group of age is a priority, as they showed the lower percentages in vaccination and may have a better response to immunization 30.

It is required to combine efforts for educating health personnel, managers responsible for making public health decisions and the general public about the advantages and benefits of immunization of the elderly, especially with the arrival of the herpes zoster vaccine. Public health programs for vaccination are overdue for integrally including older adults in their main goals, with the same benefits currently implemented for the pediatric population, in order to obtain significant consequences of prevention.

Finally, it is necessary to conduct studies aiming at measuring the impact of prevention at the clinical, economic and social level.

## Conclusion

Vaccination campaigns should be strengthened to increase vaccination coverage, especially in the group more reticent to vaccination or vulnerable to reach it such as the disabled elder.
